# Towards a Novel Monitor of Intraoperative Awareness: Selecting Paradigm Settings for a Movement-Based Brain-Computer Interface

**DOI:** 10.1371/journal.pone.0044336

**Published:** 2012-09-06

**Authors:** Yvonne M. Blokland, Jason D. R. Farquhar, Jo Mourisse, Gert J. Scheffer, Jos G. C. Lerou, Jörgen Bruhn

**Affiliations:** 1 Radboud University Nijmegen Medical Centre, Department of Anaesthesiology, Pain and Palliative Medicine, Nijmegen, The Netherlands; 2 Radboud University Nijmegen, Donders Institute for Brain, Cognition and Behaviour, Nijmegen, The Netherlands; Weill Cornell Medical College, United States of America

## Abstract

During 0.1–0.2% of operations with general anesthesia, patients become aware during surgery. Unfortunately, pharmacologically paralyzed patients cannot seek attention by moving. Their attempted movements may however induce detectable EEG changes over the motor cortex. Here, methods from the area of movement-based brain-computer interfacing are proposed as a novel direction in anesthesia monitoring. Optimal settings for development of such a paradigm are studied to allow for a clinically feasible system. A classifier was trained on recorded EEG data of ten healthy non-anesthetized participants executing 3-second movement tasks. Extensive analysis was performed on this data to obtain an optimal EEG channel set and optimal features for use in a movement detection paradigm. EEG during movement could be distinguished from EEG during non-movement with very high accuracy. After a short calibration session, an average classification rate of 92% was obtained using nine EEG channels over the motor cortex, combined movement and post-movement signals, a frequency resolution of 4 Hz and a frequency range of 8–24 Hz. Using Monte Carlo simulation and a simple decision making paradigm, this translated into a probability of 99% of true positive movement detection within the first two and a half minutes after movement onset. A very low mean false positive rate of <0.01% was obtained. The current results corroborate the feasibility of detecting movement-related EEG signals, bearing in mind the clinical demands for use during surgery. Based on these results further clinical testing can be initiated.

## Introduction

In 0.1–0.2% of surgeries involving general anesthesia, patients experience unintended intraoperative awareness [1]. Two more recent studies report even higher incidences of 1% and 0.4% [2], [3]. The phenomenon is frequently described [4]–[6] and several monitors of depth of anesthesia (e.g. Bispectral Index; Entropy Module) are now in clinical use. Nevertheless, current technology cannot prevent awareness in every patient.

Up until now, development of electroencephalography (EEG)-based monitors of depth of anesthesia has focused on general state changes in the EEG caused by administration of anesthetic drugs. As clear neural correlates of consciousness have not yet been found, current measures rely on neural processes that are unclear and cannot be properly controlled. Contrastingly, in Brain Computer Interface (BCI) research, mental tasks with clear neural responses are used as input to control a device or provide other means of communication without the use of overt behaviour. BCIs decode information from brain activity, commonly measured by EEG, and convert this information to a sensible output, e.g. a command for the connected device or computer [7]. Potential users of BCIs are, for instance, patients suffering from neurodegenerative diseases such as Amyotrophic Lateral Sclerosis, where despite severe motor impairment, cognitive functioning usually remains largely intact. BCIs circumvent the route of overt movement and directly interpret the user’s intentions from the brain signal.

A commonly used paradigm is the movement-based BCI. It uses the mu rhythm activity (8–12 Hz) in the sensorimotor cortex and the related 18–25 Hz beta rhythm. These have been found to decrease in amplitude during movement or planning of movement (event-related desynchronization or ERD) and increase right after movement (event-related synchronization or ERS) [8]. This holds for executed as well as imagined movement. So, by detecting ERD and/or ERS, the BCI can infer that the user was executing or imagining a certain movement.

Patients have consistently reported trying to move when they became aware during surgery [4], [9]. However, neuromuscular blocking agents may entirely prevent the patient to move. The above evidence shows that movement, whether covert or overt, has very clear neural correlates that can be detected with high reliability. We propose a monitor relying on these specific correlates to detect attempted movement, and therefore awareness, during anesthesia.

The specific system settings required for a feasible clinical paradigm have not yet been determined. Numerous parameters influence the setup and performance of a BCI, depending on the desired application, while each application also has its own requisites. Most importantly, the paradigm presented here requires 1) a small, generic electrode set, thus ensuring a short setup time, and 2) a minimal false positive rate (FPR). False positives occur when the system detects a ‘hit’ when attempted movement is in fact absent. Contrastingly, false negatives occur when the system fails to detect the presence of an attempted movement. Therefore, they increase the reaction time of the system as several attempts may be required for a correct detection. Although for some applications speed may be prioritized over accuracy, for the current application it is important that false alarms are kept to an absolute minimum. Considering that anesthetists are obliged to remain focused at all times, more than one false alarm in two to three hours operating time and a delay of more than 2.5 minutes to detect attempted movement do not seem to be clinically acceptable.

Here, we first investigate what EEG frequency resolution, frequency range, EEG channels and which movement-related brain signals provide the most reliable information for possible intraoperative use. Then, in order to explore the feasibility of this paradigm in clinical situations, these optimized settings are used in a simulation of a running system, showing that the resulting false positive rate and true positive rate meet our clinical requirements.

## Materials and Methods

### Ethics Statement

The protocol was approved by the Ethical Committee of the Faculty of Social Sciences of the Radboud University Nijmegen. All subjects gave written informed consent and procedures were according to the Declaration of Helsinki.

### Participants

The electroencephalogram of ten healthy, non-anesthetized and non-paralyzed participants (24–44 yrs, 4 males) was obtained while they were performing a series of movement tasks. All participants had normal or corrected-to-normal vision and hearing and none had any known neurological impairments.

### Materials and Procedure

**Figure 1:**
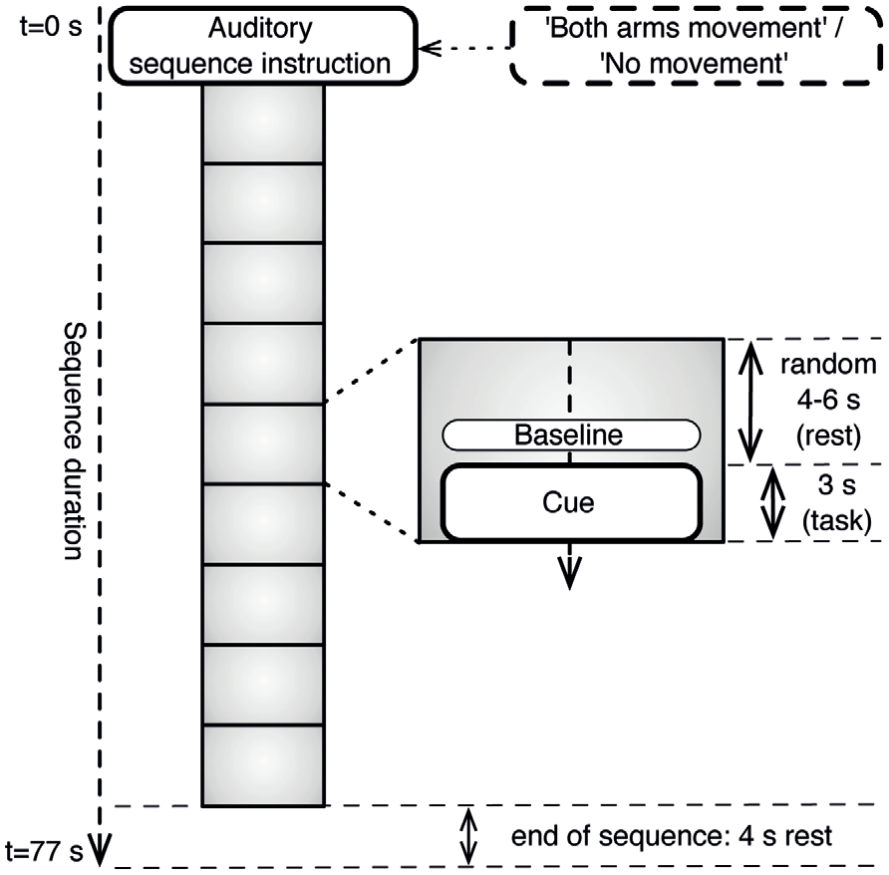
Stimulus sequence. Each sequence started with an auditory sequence instruction, i.e. ‘No Movement’ or ‘Both Arms Movement’. Following the instruction were nine trials consisting of a cue (task) and their corresponding baseline periods used for analysis.

Sequences of nine consecutive movement trials were presented to the subjects. Each trial consisted of an auditory 3-second cue preceded by a silence interval of a random duration between 4 and 6 seconds (Figure 1). At the start of each sequence, an auditory instruction was given explaining the task for the upcoming trials. The three types of sequence instruction were ‘Right Hand Movement’, ‘Both Arms Movement’ and ‘No Movement’. As the comparison between classifying either ‘Right Hand’ or ‘Both Arms’ movement has been reported elsewhere [10] and no major differences were found, the current paper only takes into account the ‘Both Arms Movement’ condition as compared to the ‘No Movement’ condition.

In the ‘Both Arms Movement’ condition subjects made a gross movement with both arms during the auditory cues. In the ‘No Movement’ condition subjects were instructed to keep still. Participants were asked to keep their eyes closed throughout the entire sequence. However, visual instructions were displayed at all times in case the subject needed a quick reminder of what the task was for that particular sequence. The experiment was self-paced, i.e. between sequences participants could have a short rest and start the next sequence themselves by pressing a button.

In total, 144 trials were collected per condition, equally divided over four experimental blocks, i.e. each block containing 4 sequences of 9 trials for each movement type. Within each block, presentation of the sequences was randomized. A short practice block to get the participants acquainted with the task preceded the actual measurements.

A Biosemi Active-2 system was used for EEG recording of 64 channels placed according to the 10/20 system [11], sampled at 2048 Hz and then downsampled to 256 Hz. Additionally, EMG of the right arm was recorded. Electrode offsets were kept below 25 µV before starting the measurement. Stimuli were played through passive speakers (Monacor, type MKS-28/WS) at a comfortable listening level. Additional instructions were displayed on a 17^2^ TFT display with a resolution of 600×800. The experiment was programmed in and run on the BrainStream platform (http://www.brainstream.nu) version 1.0, i.e. a Matlab (MathWorks Inc., MA, USA) toolbox especially developed for performing online BCI-experiments, using Psychtoolbox (http://psychtoolbox.org) for stimulus presentation.

### Analyses

A grand average time-frequency plot using a baseline from −1.5 to −0.5 seconds before the start of the task was computed to define the onset and offset of the event-related desynchronization. Based on this plot, trials used for classification were fixed at a period lasting from movement onset (t = 0 s) until one second after movement offset (t = 4 s). As a post-movement synchronization was also visible in the time-frequency plot, classification rates were computed for the post-movement period (t = 4–6 s) as well. From now on, the period from 0–4 seconds will be referred to as the ‘ERD period’ and the period from 4–6 seconds as the ‘ERS period’. To remove slow drift, linear detrending was performed. After calculating the surface Laplacian reference for each channel using Perrin’s spherical spline interpolation method [12], the power spectral density was computed for 8–24 Hz using Welch’s method [13] with a hanning taper applied to 50% overlapping windows. We also investigated which were the optimal spectral features in terms of frequency resolution and range, by varying the size of the window (125, 250, 500, or 1000 ms) and varying the subset of frequencies in the mu/beta range to keep for further analysis. This subset of power spectral features for each selected channel was then used to train a quadratically-regularized linear logistic regression classifier (rLLR) [14] to distinguish between each subject’s specific pattern of spatial and spectral activation for the ‘Both Arms Movement’ and ‘No Movement’ tasks. This classifier uses a subset of the data to find a linear weighting over the input features which gives the best fit to the data (i.e. most accurate predictions), subject to a regularization constraint that excludes unlikely values, e.g. extremely large weights for any feature. What distinguishes rLLR from other similar classifiers (such as Support Vector Machines [15]), is that rLLR gives a natural estimate of the class probability,
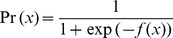
where *x* is the set of input features, *f*(*x*) is the classifier’s decision value found by making a weighted summation of the input features *x* and the classifier’s weighting *w* over these features, with an offset *b*, i.e.



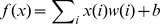



Thus, although not used in the present work, with rLLR one obtains a natural estimate of the classifier’s confidence in its prediction, which is valuable in a monitoring style application such as the one studied here. The classifier’s performance was estimated using ten-fold cross-validation, thus creating ten non-overlapping test sets. After computing classification results for the ERD period and the ERS period separately, results were also computed for a classifier trained with features from both periods.

**Figure 2:**
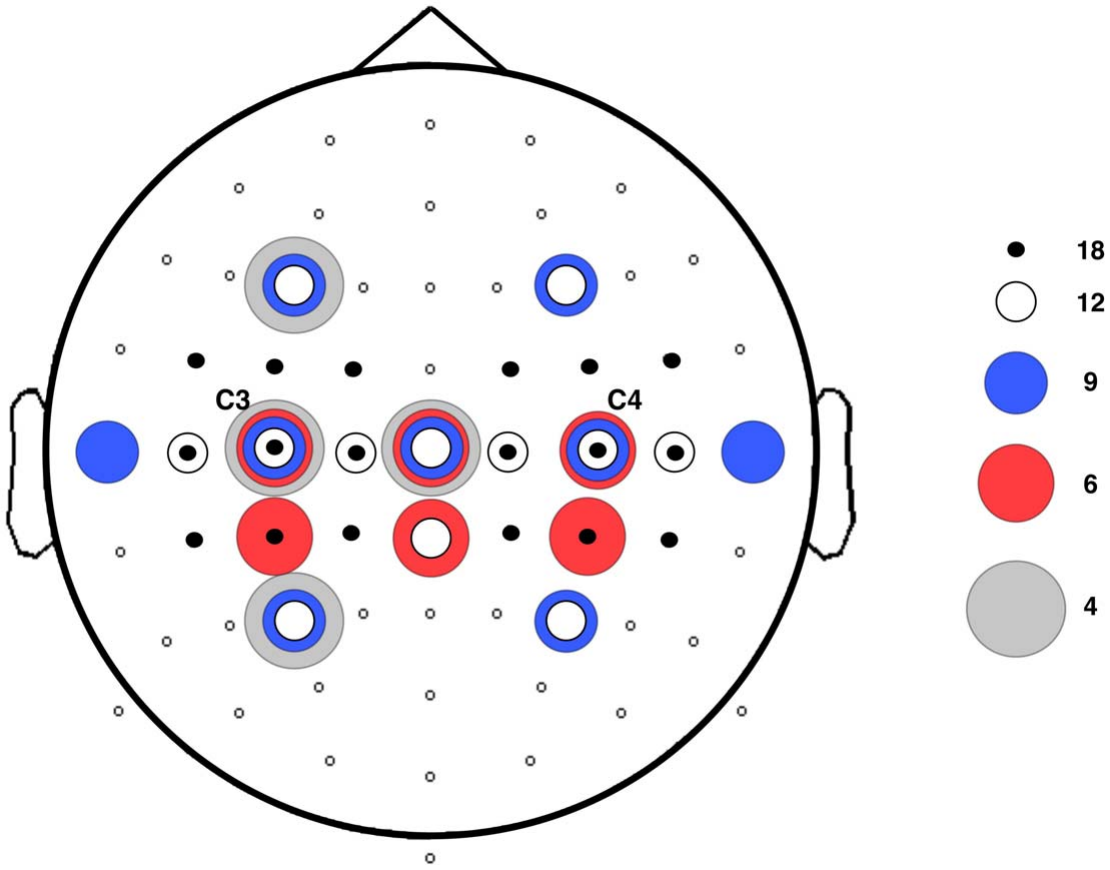
EEG Electrode positions used for analysis. The full set of 64 electrodes was used for computation of the grand average time-frequency plot. The remaining channel sets consisted of 18, 12, 9 and 6 channels. The 4-channel set denotes the Laplacian C3.

A total of six EEG channel combinations was tested in order to find an optimal balance between setup time and accuracy. Classification results obtained with the use of all channels were compared to the results of using an 18-channel set [16], as well as a 12-, 9- and 6-channel set. The positioning of the electrodes for each set is shown in Figure 2. As the ERD and ERS are especially prevalent in the motor cortex, channel sets all surrounded C3 and C4. Furthermore, the use of a Laplacian C3 [17] was evaluated and its performance was compared to that of the other channel sets. In these analyses, removal of all channels not part of the relevant channel set was done prior to all other preprocessing steps.

The 9-channel set was used to check for possible further optimization. First, in addition to the 4 Hz frequency resolution, i.e. creating one feature in the frequency dimension for each 4 Hz window, classification rates were calculated using resolutions of 1, 2 and 8 Hz. Second, using a resolution of 4 Hz, classification rates were calculated for the additional frequency ranges of 8–20 Hz and 8–28 Hz.

Next, classification rates were recomputed to simulate a clinical scenario with a 10–15 minute pre-operative calibration session for classifier training. Here, we used the first experimental block for classifier training (‘calibration’) and then calculated classification rates for the remaining three blocks (‘operating time’). Results were compared with the results of the ten-fold cross-validation and calculated for the 12-, 9- and 6-channel sets.

The classification results after training on one block, with the 9-channel set and the combined ERD and ERS periods, were used in a simulated decision making paradigm. In this decision-making paradigm, four consecutive positive classifications (i.e. ‘movement’) would be required for the monitor to give the corresponding warning.

Given the above assumptions, the cumulative probability of a true positive detection after an increasing number of movement trials was calculated. As each experimental sequence consisted of nine trials, this cumulative probability could be determined from the collected data up to nine trials (72 seconds) for each subject. To further extend this period, another nine trials were simulated using a Monte Carlo method [18].

The false positive rate was also determined, with a similar requirement of four consecutive positive classifications in a row, calculating it as *(1-classification rate)^4^*. This number represents the average duration of monitoring before a false positive event will occur.

All software was developed ‘in-house’ using Matlab, and is available as part of the Brainstream platform or directly from the authors on request.

## Results

**Figure 3:**
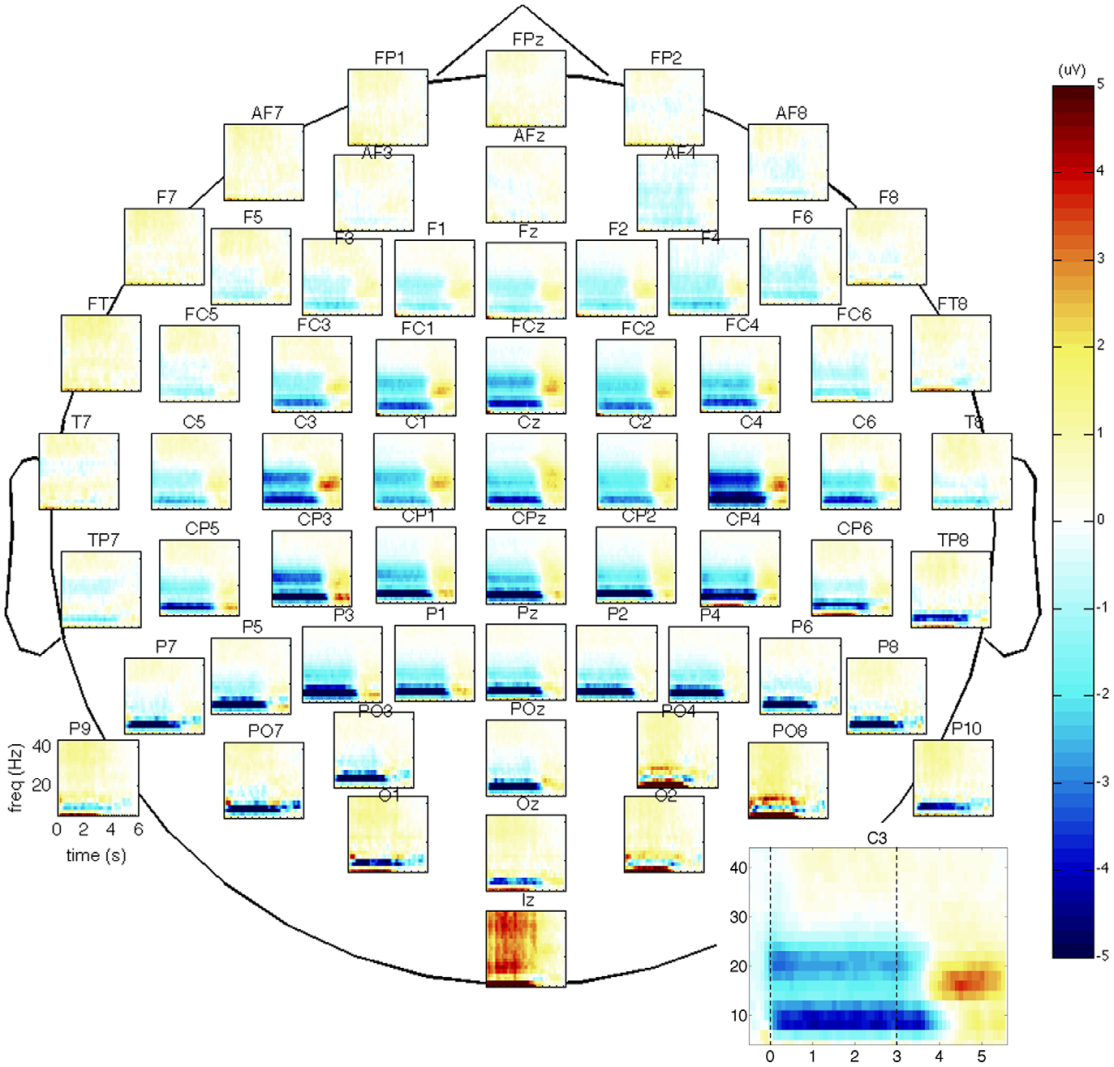
Grand average time-frequency plot of all 64 channels. Spatial downsampling was performed with a Surface Laplacian; the period from t = −1.5 to t = −0.5 s was used as the baseline period. Blue colouring represents ERD; red represents ERS. The motor cortex is situated in the central regions (C3–C4). An enlargement of channel C3 is shown in the right-hand corner, with the dashed lines indicating the onset and offset of the auditory cue (task period).

The average time frequency plot of the ‘Both Arms Movement’ trials of all participants is given in Figure 3. A clear power decrease during the movement period (ERD) and power increase after movement offset (ERS) are seen, especially in the channels located over the motor cortex. For example, in channel C3, in the 10–14 Hz range the mean power values for the ERD period decreased with 30.4% (SE 0.9%) during ‘Both Arms Movement’ as compared to ‘No Movement’, whereas in the 14–18 Hz range mean values for the ERS period increased with 27.4% (SE 2.1%) during ‘Both Arms Movement’ as compared to ‘No Movement’. An extended ERD period after task offset is visible. The corresponding extended muscle activity observed in the participants’ EMG data suggests this can most likely be attributed to task response time.

**Figure 4:**
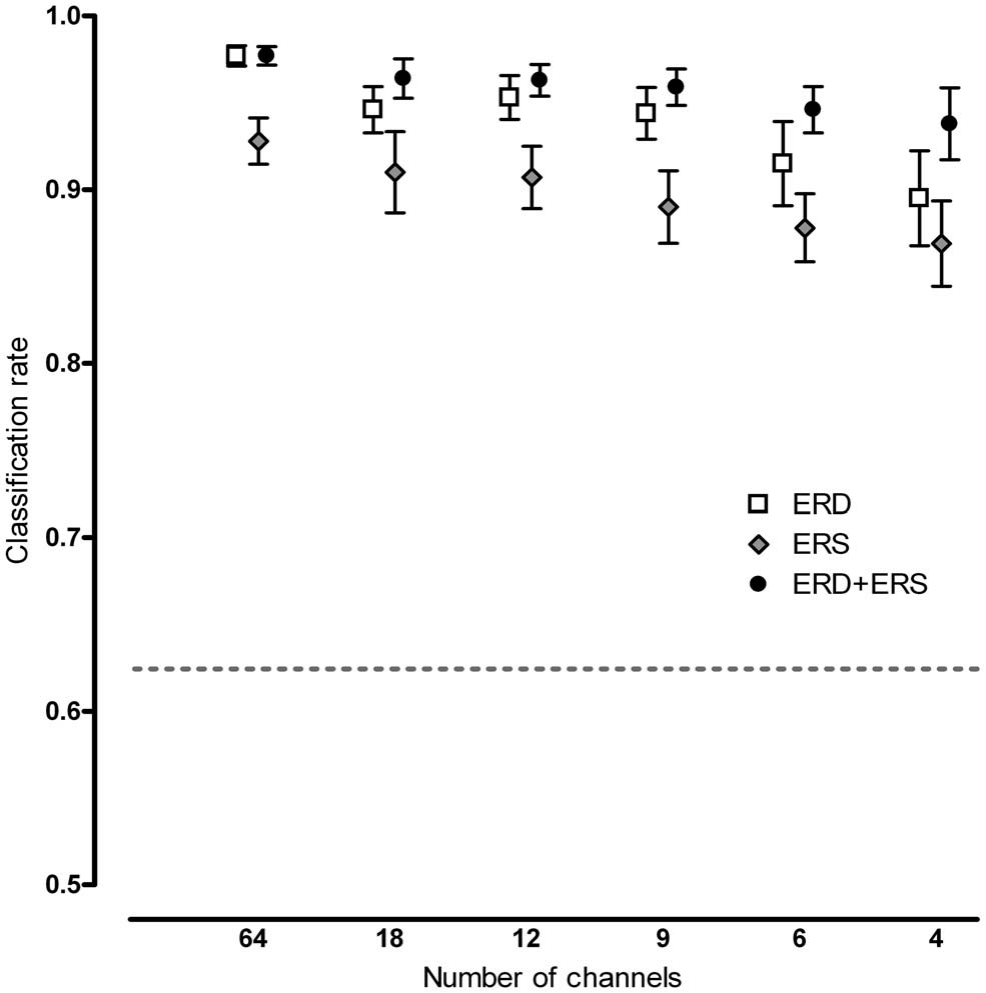
Decrease in average classification rate with corresponding standard errors when reducing the number of trials. Information was used from either t = 0–4 s (ERD), t = 4–6 s (ERS) or both (ERD+ERS). Channel sets correspond to the channel sets in Figure 2. To clearly show the rates for all time periods, data points are slightly shifted to either left or right. The dashed line represents the binomial confidence interval, i.e. all classification rates above this line are significantly better than chance (p = 0.01).

The classification rate decreased when reducing the amount of channels (Figure 4). Single trial results are shown using only the ERD or ERS period as well as using them simultaneously. For the ERD period, the average classification rate was 98% when using all 64 channels, whereas for the 18-, 12- and 9-channel sets the rates were all approximately 95%. When reducing further to a 6- and 4-channel set, rates decreased to 92% and 90%, respectively.

Although the post-movement period between 4 and 6 seconds, associated with ERS, gave slightly lower classification performance than the information from ERD *during* movement, combining these results further increased single trial results. The 18-, 12- and 9-channel sets yielded an average rate of 96%, the 6-channel set yielded 95% and the Laplacian C3 (4 channels) benefited most from incorporating the ERS feature, with rates increasing to 94%.

Tables 1 and 2 show that the use of different frequency resolutions and frequency bands yielded nearly unchanged classification results.

**Table 1:** Single trial classification rates per subject using different frequency resolutions.

Subject no.	1 Hz	2 Hz	4 Hz	8 Hz
1	0.99	0.98	0.99	0.99
2	0.94	0.95	0.94	0.96
3	0.89	0.87	0.89	0.87
4	0.96	0.96	0.97	0.95
5	0.93	0.94	0.95	0.93
6	0.98	0.98	0.98	0.98
7	0.92	0.92	0.94	0.92
8	0.98	0.99	0.99	0.99
9	0.97	0.98	0.98	0.98
10	0.99	0.99	0.98	0.98
Average	0.96	0.96	0.96	0.96

Calculations were done using 9 channels and the combined ERD and ERS periods.

**Table 2:** Single trial classification rates per subject using different frequency ranges.

Subject no.	8–20 Hz	8–24 Hz	8–28 Hz
1	0.99	0.99	0.99
2	0.97	0.94	0.94
3	0.9	0.89	0.89
4	0.96	0.97	0.97
5	0.93	0.95	0.96
6	0.98	0.98	0.98
7	0.93	0.94	0.94
8	0.98	0.99	0.98
9	0.98	0.98	0.99
10	0.98	0.98	0.98
Average	0.96	0.96	0.96

Calculations were done using 9 channels and the combined ERD and ERS periods.

Compared to the classification results when using ten-fold cross-validation over all collected data, results of the three final blocks with the first block used as training session were slightly lower (Table 3).

**Table 3:** Classification rates using ten-fold cross-validation (10-fold) versus using only the 1^st^ experimental block (1^st^ block) for training.

No. of channels	Time period	10-fold	1st block
12	ERD	0.95	0.87
12	ERD+ERS	0.96	0.91
9	ERD	0.94	0.85
9	ERD+ERS	0.96	0.92
6	ERD	0.92	0.86
6	ERD+ERS	0.95	0.9

**Figure 5:**
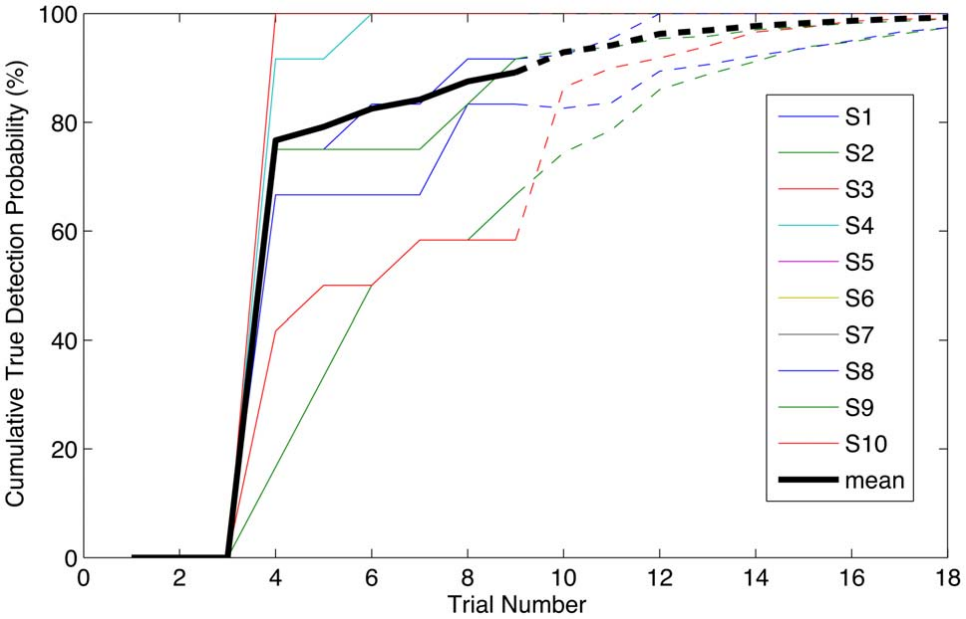
Cumulative probability of true positive monitor output after start of movement. Movement was assumed to start at trial 1 (t = 0 s) with a trial duration of 8 seconds. As in this paradigm 4 positive classifications in a row are needed for a positive monitor output, the first possible positive monitor output is at trial 4 i.e. 32 seconds after movement onset. For each subject plus the average of all subjects, the solid line shows the output for the recorded sequences. The dashed lines show the interpolation of this output for another 9 trials using a Monte Carlo simulation.

In Figure 5 the results of the mathematical simulation of the decision making paradigm are shown, in which 4 trials in a row classified as ‘Both Arms Movement’ define a positive monitor output. The cumulative probability of a true positive monitor output with an increasing delay after movement onset was calculated. These results are for a classifier trained on 1 block of data only (10–15 minutes), using the 9-channel set and both the ERD and ERS periods. Actual performance was calculated for the 9-trial sequences, results for the remaining 9 trials were obtained by means of a Monte Carlo simulation.

The calculated rate of a false positive monitor output, using similar settings, with 4 trials in a row falsely classified as ‘Both Arms Movement’ during ‘No Movement’, is ≤ 0,03% in all subjects with a mean of <0.01%. This translates into an average duration until a false positive event occurs of >7 hours in all subjects.

## Discussion

The event-related desynchronization (ERD) and -synchronization (ERS) accompanying movement are features commonly used in movement-based Brain-Computer Interface paradigms. A new BCI application, based on these features, has been proposed here: detection of attempted movement to signal intraoperative awareness. A classifier was trained to distinguish movement trials from non-movement trials based on frequency information in the EEG. We tested several paradigm settings in order to find an optimal combination of factors contributing to the BCI, therefore allowing for a system that is clinically feasible.

The principal finding of this study is that a single trial classification of 92% is obtained after a short training period, with only nine EEG channels if the ERD and ERS features are used simultaneously, as well as a 4 Hz frequency bin and a 8–24 Hz frequency range. With the requirement of four trials in a row classified as positive before a positive monitor output is generated, this translates into an extremely high probability of a true positive response within two and a half minutes after movement onset (99.3%) and a minimal false positive rate (<0.01%). Hence, important clinical requirements are met with the proposed setup: minimal setup time because of the small number of EEG channels and high system accuracy because of the strength of the signal. Although not all feature settings chosen here proved to be significantly better than others, and as such we cannot describe a true optimal set as yet, the best configuration found in this study was sufficient to meet our requirements with regard to intraoperative use.

Although using 32 to 64 channels for classification is common in BCI research, there are numerous reasons for reducing the number of channels used: reduction of EEG preparation time, reduction of the cost of the acquisition hardware and a reduced risk of overfitting the classifier [16]. Rather than aiming to find a minimal electrode set for individual subjects [16], [19], we are interested in a generic minimal electrode set that is feasible for all users.

A major reduction in EEG setup time was obtained at a very low cost in terms of information loss. An optimized balance between the number of channels and system accuracy was obtained using a set of nine channels at electrode positions C3, C4, Cz, F3, F4, P3, P4, T7 and T8. After this major reduction in channel set size, i.e. from 64 to 9 channels, single trial classification rates decreased only slightly from 98% to 96%. Further reducing the amount of channels does not decrease EEG setup time by much whereas classification rates are still reduced. Recently, even single Laplacian channel setups have been discussed (using Laplacian C3 to detect hand movement [17] and using Laplacian Cz to detect foot movement [20]). However, this type of channel setup still requires three to four surrounding electrodes in order to be able to make the Laplacian derivation. In our study a rate of 90% was obtained for the Laplacian C3 set (4 channels) when using only the ERD period and of 94% when using the ERD and ERS periods combined.

No major differences were found between different frequency resolutions. One might expect generally larger frequency resolutions to be too coarse to specifically capture the movement-induced changes in a specific frequency band of the power spectrum, whereas generally smaller frequency resolutions might have the risk of overfitting the classifier. However, these effects cannot be directly derived from our findings.

Our analysis showed that leaving out frequencies above 24 Hz, and even above 20 Hz, does not decrease classification results, implying that frequencies in the upper beta-band/lower gamma-band do not contribute significantly to the classifiability of the signal. Whereas typically movement-induced changes in the alpha- and beta-band are used in BCI algorithms, changes in higher frequency bands (i.e. gamma) have also been used [21], [22]. It has been debated whether the higher frequency ranges can actually show cognitive processing or whether they mostly represent EMG. Whitham and colleagues [23] showed that frequency ranges above 25 Hz are largely contaminated by EMG and therefore do not necessarily give much information about EEG signatures. In that particular study, EEG obtained from participants temporarily paralyzed with cisatracurium was compared to data from non-paralyzed individuals. Signals above 25 Hz were hugely reduced in power after paralysis, even in central scalp regions. In the current paper, paralysis is precisely the problem we are addressing; hence we need to make sure that our information is not contaminated by EMG. Therefore, the frequency bands used for analysis were reduced down to 24 Hz, with the results suggesting that we are in fact detecting EEG, not EMG.

The use of awareness monitors based on spontaneous EEG, such as the Bispectral Index or Entropy Module, does not decrease the rate of intraoperative awareness as opposed to simply keeping the endtidal concentration of volatile anesthetics above 0.7 MAC [24]. These monitors do not measure signs of consciousness but instead a pharmacodynamic effect of anesthetic drugs on the spontaneous EEG. Even worse, their ability to help titrating anesthetic drugs during general anesthesia has been questioned recently [25]. We therefore propose a completely new paradigm to detect intraoperative awareness, based on movement-related BCIs. The main underlying problem of intraoperative awareness is the fact that pharmacologically paralyzed patients under general anesthesia are unable to move and thus cannot seek attention from the anesthesiologist or surgeon. Their attempted movements, consistently described in the awareness literature, would now be translated into a monitor output by an algorithm detecting the movement-induced EEG changes over the motor cortex.

Ideally, such a BCI-based system should be asynchronous allowing the detection of movement at any time. However, despite of their user benefits, asynchronous systems are not widely used in BCI. Their lack of a time lock makes the analysis of the EEG signal more challenging. Here, we propose a cued synchronous design, meaning that patients would be instructed to (try to) move their hands/arms during sounds, continuously played throughout the operation, and not to move during the silence periods. Prior to anesthesia and surgery, there would be a 10–15 minute calibration session in order for the patient to get acquainted with the task and to train the classifier on the patient’s signals. During the operation, detection of the attempted movements would activate the BCI-based awareness monitor. It might be argued that the need for a 10-minute training session before an operation is prohibitive. Recent results in the movement-based BCI literature on so called ‘zero training’ BCIs indicate that with more advanced signal classification methods this training period may not be required [26]. This is an area for future research.

Low doses of anesthetic drugs might be present during cases of awareness. Their effect on movement-induced EEG changes is still unknown. It might be questioned whether patients under anesthesia who are somewhat conscious are able to attend well to a task demand of only trying to move when a sound is being played. Further research is required to test the influence of anesthetic drugs on both people’s ability to perform such a task and the corresponding brain signal.

Although in this study actual executed movements were used, eventually we are interested in studying the EEG effects of attempted but pharmacologically blocked movements. Nikulin and colleagues [27] showed that so-called ‘quasi movements’, intended movements deliberately minimized to such an extent that they become undetectable with EMG, generate a similar but stronger response than imagined movements and therefore qualify as an improved task for BCI over traditional covert movements. Likewise, attempted movements from individuals paralyzed after stroke have been found to be detectable from EEG [28].

Despite the abundance of literature on the choice of parameter settings in BCI, there is no widely accepted standard BCI procedure available that can be used unaltered for monitoring of attempted movements during anesthesia. Here, a systematic analysis of such settings was carried out, bearing in mind the specific clinical requirements. Important aspects of this study adding to the field and bringing us closer to the use of this approach in anesthesia are.

1. A generic, subject independent subset of electrodes resulting in very high classification performance in each individual subject. This adds to the scarce data about subject independent channel selection [29], whereas commonly BCI studies focus on individually optimized channel selection since this is most interesting for the long-term use in neurological patients. For clinical use during anesthesia however, a standard montage of a reduced electrode set, working for all patients, is required. Here we have given a systematic comparison between different electrode sets and shown that the 9 channels mentioned above are sufficient to obtain the true positive and false positive rates required in the context of anesthesia monitoring.
2. The fact that the maximum frequency considered can be lowered down to 24 Hz and even 20 Hz without significantly decreasing classification performance. Whereas in classical BCI paradigms this may be of lesser importance, as conditions during the training period are largely comparable to those during the testing phase, in our case any EMG activity that may be present in the pre-operative training session will certainly be absent after administration of neuromuscular relaxant drugs.
3. The novel suggestion of a “four-in-a-row” algorithm for decision making, proving to be appropriate for an intended very low false positive rate (0.01%) and a high true positive rate (99% within 2.5 minutes). Based on our experience in clinical anesthesia and anesthesia monitoring, the maximum acceptable rate of false alarms is one per 2 hours operating time, with a delay of no more than 2.5 minutes before detection of attempted movement. These assumptions can be used as blueprints for other BCI groups without a clinical anesthesia background to further improve the suggested concepts.

In conclusion, a highly accurate system has been proposed that, despite its current limitations, can be further developed into a BCI monitor to detect intraoperative awareness in a clinical environment. Future work will test this paradigm in temporarily paralyzed participants and in the presence of low doses of anesthetics.
